# Associations between Central Obesity and Outcomes of Adult In-hospital Cardiac Arrest: A Retrospective Cohort Study

**DOI:** 10.1038/s41598-020-61426-z

**Published:** 2020-03-12

**Authors:** Chih-Hung Wang, Wei-Tien Chang, Chien-Hua Huang, Min-Shan Tsai, Tsung-Chien Lu, Eric Chou, Yen-Wen Wu, Wen-Jone Chen

**Affiliations:** 1 Department of Emergency Medicine, National Taiwan University Hospital, Taipei, Taiwan; 2 Department of Emergency Medicine, College of Medicine, National Taiwan University, Taipei, Taiwan; 3 Department of Emergency Medicine, Baylor Scott & White All Saints Medical Center, Fort Worth, Texas USA; 4 Departments of Internal Medicine and Nuclear Medicine, National Taiwan University Hospital and National Taiwan University College of Medicine, Taipei, Taiwan; 5 Department of Nuclear Medicine and Cardiology Division of Cardiovascular Medical Center, Far Eastern Memorial Hospital, New Taipei City, Taiwan; 6 National Yang-Ming University School of Medicine, Taipei, Taiwan; 7 Division of Cardiology, Department of Internal Medicine, National Taiwan University Hospital and National Taiwan University College of Medicine, Taipei, Taiwan

**Keywords:** Cardiology, Endocrinology, Medical research

## Abstract

To investigate the association between central obesity and outcomes following in-hospital cardiac arrest (IHCA). A single-centred retrospective study was conducted. Adult patients that experienced IHCA during 2006–2015 were screened. Body mass index (BMI) was calculated at hospital admission. Central obesity-related anthropometric parameters were measured by analysing computed tomography images. A total of 648 patients were included, with mean BMI of 23.0 kg/m^2^. The proportions of BMI-defined obesity in this cohort were underweight (13.1%), normal weight (41.4%), overweight (31.5%) and obesity (14.0%). The mean waist circumference was 85.9 cm with mean waist-to-height ratio (WHtR) of 0.53. The mean sagittal abdominal diameter was 21.2 cm with mean anterior and posterior abdominal subcutaneous adipose tissue (SAT) depths of 1.6 and 2.0 cm, respectively. Multivariate logistic regression analyses indicated BMI of 11.7–23.3 kg/m^2^ (odds ratio [OR]: 2.53, 95% confidence interval [CI]: 1.10–5.85; p-value = 0.03), WHtR of 0.49–0.59 (OR: 3.45, 95% CI: 1.56–7.65; p-value = 0.002) and anterior abdominal SAT depth <1.9 cm (OR: 2.84, 95% CI: 1.05–7.74; p-value = 0.04) were positively associated with the favourable neurological outcome. Central obesity was associated with poor IHCA outcomes, after adjusting for the effects of BMI.

## Introduction

Each year, approximately 209,000 American patients experience in-hospital cardiac arrest (IHCA)^1^. About 24% of IHCA patients survive to hospital discharge; among them, 14% experience significant neurological disability^1^. The prevalence of obesity has increased rapidly over the last few decades with 40% of the American population classified as obesity^2^.

Body mass index (BMI) has been demonstrated to be associated with IHCA outcomes^3,4^. Jain *et al*^3^. reported that for IHCA with shockable rhythms, patients with BMI < 18.5 kg/m^2^, BMI 18.5–24.9 kg/m^2^ or BMI ≥ 35 kg/m^2^ had lower survival rates compared with patients with BMI 25.0–29.9 kg/m^2^ or BMI 30.0–34.9 kg/m2. However, for IHCA with non-shockable rhythms, patients with a moderately elevated BMI seemed to have better IHCA outcomes.

This so-called “obesity paradox” was also observed in other cardiovascular diseases^5^. However, using BMI as the sole obesity index does not consider body adipose distribution. In patients with coronary disease, Coutinho *et al*^6^. noted that being overweight or obese per BMI criteria did not cause increased mortality in the absence of central obesity. When parameters defining central obesity were taken into account, the phenomenon of “obesity paradox” or “BMI paradox” was less obvious^6^.

Accumulation of abdominal adipose tissue has been identified as a major cardiometabolic risk factor promoting the production of pro-inflammatory cytokines and adipokines^7^. Following resuscitation from IHCA, patients would suffer from post-cardiac arrest syndrome with generalized activation of immunologic pathways and increased risk of multiple organ failure^8^, which may be compounded by the presence of central obesity. Several anthropometric measures have been developed to describe the extent of central obesity, such as waist circumference (WC)^9^, waist-to-height ratio (WHtR)^10^ and sagittal abdominal diameter (SAD)^11^. Accordingly, in the current analysis, we attempted to investigate the association between central obesity based on these anthropometric parameters and IHCA outcomes.

## Materials and Methods

### Setting

We conducted this retrospective cohort study in a tertiary medical centre, the National Taiwan University Hospital (NTUH). A total of 2,600 beds were available in the NTUH, including 220 beds in intensive care units (ICUs). This study was performed in agreement with the Declaration of Helsinki amendments and approved by the Research Ethics Committee of National Taiwan University Hospital (reference number: 201803103RINB). The requirement of informed consent was exempted before patient data were collected. In the NTUH, a code team would be activated when patients experience IHCA in the general wards. The members of a code team include a senior resident, several junior residents, a respiratory therapist, a head nurse and several ICU nurses. For patients sustaining IHCA in the ICUs, only ICU staff would be involved in cardiopulmonary resuscitation (CPR), without activation of a code team. For all IHCA patients, manual CPR is performed without resorting to mechanical CPR devices.

### Participants

We screened patients experiencing IHCA at NTUH during 2006–2015. We included patients who met the following criteria: (1) age above 18 years, (2) confirmed pulselessness with chest compressions performed for at least 2 min and (3) no documented do-not-resuscitate order before CPR. We recorded the first event only when a single patient experienced multiple cardiac arrest events during hospitalisation. We excluded patients who experienced major trauma-related cardiac arrest. We also excluded patients without necessary radiological information for measuring WC and SAD and those without body weight or height data for calculating BMI or WHtR.

### Data collection and outcome measures

We extracted the following information for each patient: age, sex, comorbidities, variables derived from the Utstein template^12^ and critical interventions implemented during CPR and after sustained return of spontaneous circulation (ROSC). We defined sustained ROSC as ROSC lasting longer than 20 min without chest compressions performed during this period. We defined CPR duration as the time interval from the initiation of the chest compression to the end of resuscitation efforts, either due to sustained ROSC or declaration of death.

**Figure 1:**
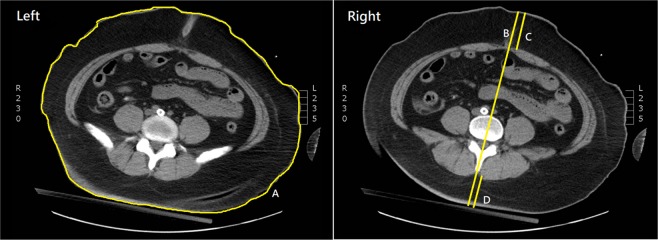
Example of measuring anthropometric parameters. Left panel: (A). waist circumference (at the umbilical level); Right panel: (B). sagittal abdominal diameter (from the anterior skin surface perpendicular to the posterior skin surface at the level of iliac crest [L4–L5]), (C). anterior abdominal subcutaneous adipose tissue depth (between the skin surface and the anterior layer of rectus sheath or linea alba), (D). posterior abdominal subcutaneous adipose tissue depth (between the skin surface and the tip of the spinous process).

We screened all computed tomography (CT) images scanned within one year prior to the IHCA event for each patient. We selected those CT images which contained the required information to measure the anthropometric parameters of interest and were the most updated study available at the time of the IHCA event. Body weight and height measured at index admission were used to calculate BMI and WHtR. The BMI was calculated as weight (kg) divided by height squared (m^2^) and classified into four categories according to World Health Organisation classification for Asian populations^13^, i.e. less than 18.5 kg/m^2^ (underweight), between 18.5 and 23 kg/m^2^ (normal weight), between 23 and 27.5 kg/m^2^ (overweight), and greater than 27.5 kg/m^2^ (obese). The WC was measured in the axial CT images at the umbilical level (cm) (Fig. 1). The WHtR was calculated as WC (cm) divided by height (cm). The SAD was measured in the axial CT images at the level of iliac crest (L4–L5) from the anterior skin surface perpendicular to the posterior skin surface (cm) (Fig. 1)^14^. Anterior and posterior abdominal subcutaneous adipose tissue (SAT) depths were also measured along the line used for measuring SAD, which were measured between the skin surface and the anterior layer of rectus sheath or linea alba anteriorly and the skin surface and the tip of the spinous process posteriorly (Fig. 1)^15^.

The primary outcome was favourable neurological status at hospital discharge; the secondary outcome was survival to hospital discharge. Favourable neurological status was defined as a score of 1 or 2 on the Cerebral Performance Category (CPC) scale^16^, which indicated sufficient cerebral function to live independently. We reviewed the relevant medical records for each patient to determine the CPC score.

### Statistical analysis

Categorical data were expressed as counts and percentages and compared using Fisher’s exact test. Continuous data were expressed as means and standard deviations and compared using Wilcoxon’s rank-sum test. A two-tailed *p*-value < 0.05 was considered statistically significant.

We selected the odds ratio (OR) as the outcome measure. The model-building procedure has been described in our previous study^4^. Briefly, we conducted multivariate logistic regression analyses to investigate the associations between independent variables and outcomes. We considered all available independent variables in the initial model-building process, regardless of whether they were counted as significant by univariate analyses. We applied the stepwise variable selection procedure using iterations between the forward and backward steps to obtain the final regression model. We set the significance thresholds for entry and to stay in the model at 0.15 to avoid excluding potential candidate variables during the variable selection process. We sequentially removed individual variables with a *p*-value > 0.05 from the interim statistical models until all regression coefficients were significant in the final model.

We used generalised additive models (GAMs)^17^ to examine non-linear effects of the continuous variables and identify the potential cut-off points for dichotomising a continuous variable during the model-building process. We tested the goodness-of-fit of the fitted regression model with *c* statistics, the adjusted generalised *R*^2^ and the Hosmer–Lemeshow goodness-of-fit test. Data were analysed using R 3.5.2 software (R Foundation for Statistical Computing, Vienna, Austria).

## Results

There was a total of 1,698 adult non-trauma IHCA patients at NTUH receiving chest compression for ≥2 min between 2006 and 2015. Among these patients, 981 of them were excluded because of a lack of necessary radiological information for measuring WC and SAD, and 69 patients were further excluded because of a lack of data on body weight or height. Finally, 648 patients were included in the analysis.

The characteristics of the cardiac arrest events are provided in Tables 1 and 2. The mean age of the included patients was 63.2 years. Their mean BMI was 23.0 kg/m^2^, mean WC was 85.9 cm and mean WHtR was 0.53. The mean SAD was 21.2 cm with mean anterior and posterior abdominal SAT depths of 1.6 and 2.0 cm, respectively. There were 278 (42.9%) and 337 (52.0%) cardiac arrest events occurring in the ICUs and on the general wards, respectively. Most of the initial rhythms (86.3%) were non-shockable rhythms, including pulseless electrical activity and asystole. The mean CPR duration was 31.1 min. There were 88 patients (13.6%) surviving to hospital discharge and 47 patients (7.3%) demonstrating favourable neurological status.

**Table 1:** Baseline characteristics of study patients.

Variables	All patients (n = 648)	Patients with favourable neurological outcome at hospital discharge (n = 47)	Patients without favourable neurological outcome at hospital discharge (n = 601)	*p*-value
Age, years (SD^a^)	63.2 (16.5)	58.1 (16.9)	63.7 (16.4)	0.03
Male, n (%)	407 (62.8)	31 (66.0)	376 (62.6)	0.75
Anthropometric parameters, (SD)				
Waist circumference, cm	85.9 (10.8)	84.4 (8.3)	86.0 (11.0)	0.30
Waist-to-height ratio	0.53 (0.07)	0.52 (0.05)	0.53 (0.07)	0.20
Sagittal abdominal diameter, cm	21.2 (3.7)	20.8 (3.1)	21.3 (3.7)	0.54
Anterior abdominal subcutaneous adipose tissue depth, cm	1.6 (0.7)	1.4 (0.5)	1.7 (0.7)	0.02
Posterior abdominal subcutaneous adipose tissue depth, cm	2.0 (1.1)	1.9 (0.9)	2.1 (1.1)	0.83
BMI, kg/m^2^ (SD)	23.0 (4.5)	21.3 (3.3)	23.1 (4.5)	0.003
Underweight, BMI < 18.5, n (%)	85 (13.1)	9 (19.1)	76 (12.6)	0.02
Normal weight, 18.5 ≦ BMI < 23, n (%)	268 (41.4)	27 (57.4)	241 (40.0)	
Overweight, 23 ≦ BMI < 27.5, n (%)	204 (31.5)	8 (17.0)	196 (32.6)	
Obese, BMI ≧ 27.5, n (%)	91 (14.0)	3 (6.4)	88 (14.6)	
Comorbidities, n (%)				
Heart failure, this admission	109 (16.8)	13 (27.7)	96 (16.0)	0.07
Heart failure, prior admission	84 (13.0)	8 (17.0)	76 (12.6)	0.37
Myocardial infarction, this admission	52 (8.0)	8 (17.0)	44 (7.3)	0.04
Myocardial infarction, prior admission	25 (3.9)	4 (8.5)	21 (3.5)	0.10
Arrhythmia	107 (16.5)	8 (17.0)	99 (16.5)	0.84
Hypotension	181 (27.9)	13 (27.7)	168 (28.0)	1
Respiratory insufficiency	450 (69.4)	24 (51.1)	426 (70.9)	0.008
Renal insufficiency	264 (40.7)	12 (25.5)	252 (41.9)	0.03
Hepatic insufficiency	151 (23.3)	6 (12.8)	145 (24.1)	0.10
Metabolic or electrolyte abnormality	110 (17.0)	4 (8.5)	106 (17.6)	0.16
Diabetes mellitus	201 (31.0)	14 (29.8)	187 (31.1)	1
Baseline evidence of motor, cognitive or functional deficits	218 (33.6)	10 (21.3)	208 (34.6)	0.08
Acute stroke	22 (3.4)	2 (4.3)	20 (3.3)	0.67
Favourable neurological status 24 h before cardiac arrest	302 (46.6)	32 (68.1)	270 (44.9)	0.002
Pneumonia	181 (27.9)	8 (17.0)	173 (28.8)	0.09
Bacteraemia	61 (9.4)	2 (4.3)	59 (9.8)	0.30
Cirrhosis	52 (8.0)	1 (2.1)	51 (8.5)	0.16
Chronic Obstructive Pulmonary Disease	36 (5.6)	3 (6.4)	33 (5.5)	0.74
Dialysis	122 (18.8)	7 (14.9)	115 (19.1)	0.56
Metastatic cancer or any blood-borne malignancy	220 (34.0)	5 (10.6)	215 (35.8)	<0.001
Charlson comorbidity index (SD)	3.3 (2.5)	2.0 (1.9)	3.4 (2.5)	<0.001

^a^SD, standard deviation. ^b^BMI, body mass index.

**Table 2:** Features, interventions and outcomes of cardiac arrest events.

Variables	All patients (n = 648)	Patients with favourable neurological outcome at hospital discharge (n = 47)	Patients without favourable neurological outcome at hospital discharge (n = 601)	*p*-value
Arrest at night, n (%)	199 (30.7)	15 (31.9)	184 (30.6)	0.87
Arrest on weekend, n (%)	182 (28.1)	12 (25.5)	170 (28.3)	0.74
Arrest location, n (%)				0.56
Intensive care unit	278 (42.9)	17 (36.2)	261 (43.4)	
General ward	337 (52.0)	27 (57.4)	310 (51.6)	
Others	33 (5.1)	3 (6.4)	30 (5.0)	
Witnessed arrest, n (%)	438 (67.6)	27 (57.4)	411 (68.4)	0.14
Monitored status, n (%)	393 (60.6)	28 (59.6)	365 (60.7)	0.88
Shockable rhythm, n (%)	89 (13.7)	17 (36.2)	72 (12.0)	<0.001
Critical care interventions in place at time of arrest, n (%)				
Mechanical ventilation	163 (25.2)	9 (19.1)	154 (25.6)	0.39
Antiarrhythmics	68 (10.5)	5 (10.6)	63 (10.5)	1
Vasopressors	270 (41.7)	17 (36.2)	253 (42.1)	0.45
Dialysis	40 (6.2)	2 (4.3)	38 (6.3)	0.76
Pulmonary artery catheter	7 (1.1)	1 (2.1)	6 (1.0)	0.41
Intra-aortic balloon pumping	5 (0.8)	1 (2.1)	4 (0.7)	0.31
CPR^a^ duration, min (SD^b^)	31.1 (28.5)	13.3 (10.9)	32.5 (29.0)	<0.001
Post-ROSC^c^ interventions, n (%)				
Extracorporeal membrane oxygenation	45 (6.9)	6 (12.8)	39 (6.5)	0.13
Targeted temperature management	2 (0.3)	0 (0)	2 (0.3)	1
Percutaneous coronary intervention	30 (4.6)	10 (21.3)	20 (0.03)	<0.001
Sustained ROSC, n (%)	383 (59.1)	47 (100)	336 (55.9)	<0.001
Survival to hospital discharge, n (%)	88 (13.6)	47 (100)	41 (6.8)	<0.001

^a^CPR, cardiopulmonary resuscitation; ^b^SD, standard deviation; ^c^ROSC, return of spontaneous circulation.

**Figure 2:**
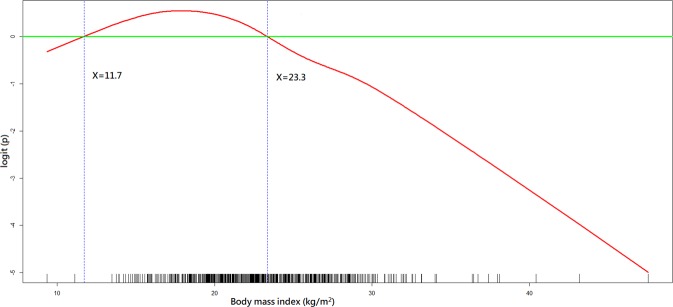
A GAM plot for the effect of body mass index on the logit of probability for the favourable neurological outcomes at hospital discharge. GAM, generalised additive model. R Core Team (2018). R: A language and environment for statistical computing. R Foundation for Statistical Computing, Vienna, Austria. http://www.R-project.org/.

**Figure 3:**
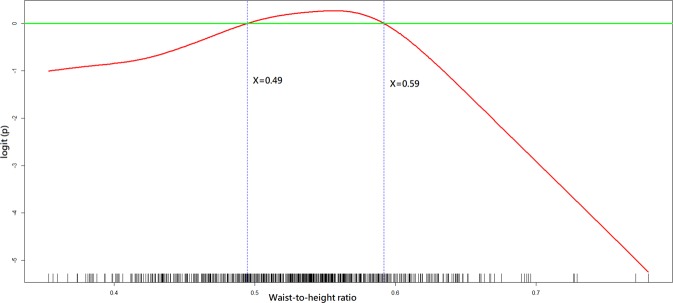
A GAM plot for the effect of waist-to-height ratio on the logit of probability for the favourable neurological outcome at hospital discharge. GAM, generalised additive model. R Core Team (2018). R: A language and environment for statistical computing. R Foundation for Statistical Computing, Vienna, Austria. http://www.R-project.org/.

**Figure 4:**
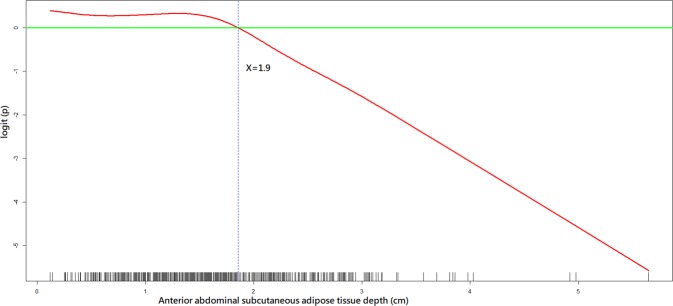
A GAM plot for the effect of anterior abdominal subcutaneous adipose tissue depth on the logit of probability for the favourable neurological outcome at hospital discharge. GAM, generalised additive model. R Core Team (2018). R: A language and environment for statistical computing. R Foundation for Statistical Computing, Vienna, Austria. http://www.R-project.org/.

As shown in Tables 1 and 2, all independent variables were put in the variable list for selection in the model-building process. As shown in Figs. 2–4, the GAM plots showed a non-linear association of logit (p), where p represented the probability for favourable neurological outcome, with BMI, WHtR and anterior abdominal SAT depth, respectively. If logit (p) was above zero, the odds for favourable neurological outcome were greater than one. Therefore, BMI, WHtR and anterior abdominal SAT depth were transformed into binary variables according to these identified cut-off points during the model-fitting process.

The BMI of 11.7–23.3 kg/m^2^ (OR: 2.53, 95% confidence interval [CI]: 1.10–5.85; *p*-value = 0.03), WHtR of 0.49–0.59 (OR: 3.45, 95% CI: 1.56–7.65; *p*-value = 0.002) and anterior abdominal SAT depth <1.9 cm (OR: 2.84, 95% CI: 1.05–7.74; *p*-value = 0.04) were positively associated with a favourable neurological outcome (Table 3). The BMI of 13.2–23.1 kg/m^2^ (OR: 2.68, 95% CI: 1.52–4.74; *p*-value < 0.001) and posterior abdominal SAT depth of 1.6–3.4 cm (OR: 2.40, 95% CI: 1.37–4.18; *p*-value = 0.002) were positively associated with survival at hospital discharge (Table 4).

**Table 3:** Multiple logistic regression model with favourable neurological outcome at hospital discharge as the dependent variable.

Independent variable^a^	Odds ratio	95% confidence interval	*p*-value
CPR^b^ duration	0.94	0.91–0.97	<0.001
Post-ROSC^c^ percutaneous coronary intervention	6.28	2.13–18.50	<0.001
Age (years)	0.97	0.95–0.99	0.001
Waist-to-height ratio of 0.49–0.59	3.45	1.56–7.65	0.002
Renal insufficiency	0.34	0.15–0.75	0.008
Metastatic cancer or any blood-borne malignancy	0.27	0.10–0.78	0.01
Body mass index of 11.7–23.3 (kg/m^2^)	2.53	1.10–5.85	0.03
Favourable neurological status 24 h before cardiac arrest	2.24	1.06–4.71	0.03
Anterior abdominal subcutaneous adipose tissue depth <1.9 (cm)	2.84	1.05–7.74	0.04
Charlson comorbidity index <4	2.99	1.03–8.61	0.04

Goodness-of-fit assessment: n = 648, adjusted generalised R2 = 0.40, the estimated area under the Receiver Operating Characteristic (ROC) curve = 0.90 and the Hosmer–Lemeshow goodness-of-fit chi-squared test p-value = 0.99.

^a^The display of independent variables is arranged in order of *p*-value.

^b^CPR, cardiopulmonary resuscitation.

^c^ROSC, return of spontaneous circulation.

**Table 4:** Multiple logistic regression model with survival to hospital discharge as the dependent variable.

Independent variable^a^	Odds ratio	95% confidence interval	*p*-value
CPR^b^ duration	0.93	0.91–0.95	<0.001
Post-ROSC^c^ percutaneous coronary intervention	6.89	2.67–17.74	<0.001
Body mass index of 13.2–23.1 (kg/m^2^)	2.68	1.52–4.74	<0.001
Respiratory insufficiency	0.38	0.22–0.67	<0.001
Hepatic insufficiency	0.26	0.11–0.61	0.002
Posterior abdominal subcutaneous adipose tissue depth of 1.6–3.4 (cm)	2.40	1.37–4.18	0.002
Metastatic cancer or any blood-borne malignancy	0.36	0.18–0.75	0.006
Charlson comorbidity index <4	2.26	1.12–4.57	0.02
Arrest at other locations	0.25	0.07–0.94	0.04

Goodness-of-fit assessment: n = 648, adjusted generalised R2 = 0.40, the estimated area under the Receiver Operating Characteristic (ROC) curve = 0.88 and the Hosmer–Lemeshow goodness-of-fit chi-squared test p-value = 0.68.

^a^ The display of independent variables is arranged by the order of *p-*value.

^b^ CPR, cardiopulmonary resuscitation.

^c^ ROSC, return of spontaneous circulation.

## Discussion

### Main findings

In this retrospective observational study, we noted that in addition to BMI, several central obesity-related anthropometric parameters were associated with IHCA outcomes, including WHtR and abdominal SAT depths. After adjusting for these anthropometric parameters, the optimal BMI range was 11.7–23.3 kg/m^2^, classified as underweight or normal weight. Among IHCA patients, the phenomenon of the “obesity paradox” disappeared after central obesity was taken into consideration. Also, even if patients were underweight or normal weight, those with central obesity may still suffer worse post-CPR outcomes.

### BMI and central obesity

Jain *et al*^3^. suggested that based on BMI, probably only underweight patients had worse IHCA outcomes but patients of overweight or obese had better outcomes. As with other cardiovascular diseases^5^, the “obesity paradox” was also observed in post-CPR patients. In contrast, our analysis indicated that patients with BMI of 11.7–23.3 kg/m^2^, categorized as underweight or normal weight based on Asian classification^13^, had better neurological outcomes. Some researches^18,19^ also revealed an association between elevated BMI and poor outcomes in post-CPR patients receiving therapeutic hypothermia. The BMI was demonstrated to be a poor discriminator of body fat and lean mass, especially in patients with cardiovascular disease^20^, which may consequently lead to these controversial observations.

In patients with coronary artery disease, Coutinho *et al*^6^. indicated that patients with normal BMI and central obesity had the worst long-term survival compared with patients with other adiposity patterns. Furthermore, they demonstrated that being overweight or obese did not cause increased mortality in the absence of central obesity. Therefore, the “obesity paradox” may simply be a “BMI paradox.” As shown in our study, when effects of central obesity were adjusted for in the statistical analysis, the optimal ranges of BMI (Fig. 2) associated with better outcomes seemed quite intuitive, rather than paradoxical.

### Anthropometric parameters for central obesity

Several commonly used anthropometric parameters for screening central obesity were investigated in the current analysis, including WHtR and WC. In comparison with WHtR, the accuracy of WC for diagnosing central obesity may decrease for tall or short individuals because height is not considered^21^. In a meta-analysis including more than 88,000 adults, Lee *et al*^22^. indicated that WHtR was the best discriminator for several cardiometabolic diseases for both men and women – superior to BMI and WC. In a Chinese study, Peng *et al*. identified that the upper limits of WHtR for discriminating cardiovascular disease with specificity above 90% were 0.55 and 0.58 for men and women, respectively^23^. Consistent with these studies^23,24^, we identified the optimal range of WHtR as 0.49–0.59, suggesting that patients with WHtR-defined central obesity may have a worse outcome.

The SAD was proposed as an alternative for estimating abdominal adipose content for patients who could not stand up to allow measuring of WC^25^. The SAD was strongly associated with cardiovascular risk^26^ and outperformed BMI in predicting mortality in ICU patients^11^. However, in our analysis, SAD was not significantly associated with IHCA outcomes. Similar to WC, SAD itself did not adjust for the factor of patient height, which may lead to underestimation or overestimation of cardiovascular risks in patients of short or tall stature. In contrast, anterior and posterior abdominal SAT depths, measured at the same level as SAD, were associated with IHCA outcomes. In comparison with WC or SAD, directly measuring the abdominal SAT depths may be more closely correlated with the extent of central obesity even though not adjusted for body height. Taken together, the significant associations of WHtR and abdominal SAT depths with IHCA outcomes may corroborate our hypothesis that central obesity, not BMI-defined obesity, was a significant risk factor for poor IHCA outcomes.

### Central obesity and IHCA outcomes

It is acknowledged that abdominal adipose accumulation has greater health consequences than peripheral obesity^27^. Increased abdominal adiposity has been demonstrated to play a critical role in the pathogenesis of metabolic syndrome^28^ and enhance the pro-inflammatory cytokine release, possibly contributing to the development of sepsis^11,29^. In a murine model, a significantly increased expression of pro-inflammatory markers, such as interleukin-6 and interleukin-1, was observed in the SAT subjected to ischaemia-reperfusion, suggesting its role as an active driver of the inflammatory response to ischaemia-reperfusion injury^30^. Among young women with central obesity, longer refilling time for microflow during the post-occlusive reactive hyperaemia response was observed, suggesting microvascular dysfunction^31^. Following resuscitation from cardiac arrest, patients may suffer from complications of post-cardiac arrest syndrome^8^. In the rat models of asphyxia-^32^ or ventricular fibrillation-induced cardiac arrest^33^, the amount of pro-inflammatory cytokines, such as interleukin-1β, had been revealed to increase after CPR. The central obesity-related pro-inflammatory response and microvascular dysfunction may thus compound these post-ROSC systemic ischaemia-reperfusion injury and inflammatory response, leading to worse outcomes. Therapeutic hypothermia (or targeted temperature management) had been shown to ameliorate these inflammatory injuries^32,33^, and thereby improve clinical outcomes^34^. However, it had been demonstrated that elevated BMI may impair the effects of therapeutic hypothermia, necessitating further investigation^18,19^. Also, statin has been shown to lower the risk of obesity-associated illnesses^35^, probably through its effect in decreasing inflammatory markers, including interleukin-1 and interleukin-6^36^. In a recent study, prior statin use was also shown to improve survival of patients following cardiac arrest^37^. Whether de novo administration of statins following ROSC would improve outcomes of patients with central obesity requires further examination.

Alternatively, the worse IHCA outcomes associated with central obesity may be explained in a mechanistic way. According to cardiac pump theory^38^, in order to provide high-quality chest compression, the rescuer’s hands should be placed on the lower half of the sternum^39^ where the maximal left ventricle diameter is assumed to be^40^. Lee *et al*^41^. evaluated the point of maximal left ventricular diameter by examining CT scans, which was noted to be more cranial in patients of obese than of normal weight. Similar to gravid uterus, central obesity may weaken and raise the diaphragm to a position higher than that for normal weight, especially when patients are in a supine position. For pregnant women in cardiac arrest, guidelines^42^ suggested that the rescuer’s hands should be positioned slightly more cranial on the sternum compared to that in non-pregnant patients. For patients with central obesity, it was probable that CPR efficiency was not optimal if the hands were placed on the lower half of the sternum^39^. In addition, thoracic anteroposterior diameter, cross-sectional area and SAT depths were also found to be associated with IHCA outcomes^4,43^. All these observations suggest that a “one-size-fits-all” strategy may not be optimal for all patients, which means that the location and depth of chest compression would be better adjusted according to patient body size and habitus to improve CPR efficiency and outcomes. Animal studies suggested that haemodynamic-directed CPR may improve IHCA outcomes because rescuers could titrate chest compression depth and vasopressor use according to patients’ haemodynamic response^44^, which may be readily applicable in ICUs because patients already had invasive physiological monitors *in situ*. Whether universal CPR techniques should be adapted for patients with central obesity as for pregnant women^42^ should be further examined.

### Study limitations

First, because this was an observational study, instead of a causal relationship, we can only establish an association between independent and dependent variables. Second, the studied cohort included heterogenous population, which may threaten the validity of the study results. Third, the real depth of chest compression could not be estimated retrospectively. The CT images were obtained in static rather than real-time dynamic CPR scenarios. Therefore, the underlying mechanisms explaining our observations may only be verified in prospective studies. Finally, despite that we have already screened the CT images performed as long as one year before the IHCA event, 57.8% of patients (981/1698) were excluded because of a lack of CT images, which may have led to selection bias. Extending the screening period may yield more available images and possibly include more patients. However, the risks would also be higher that the adiposity patterns would change significantly during this longer screening period. This limitation may only be overcome by a prospective study with these anthropometric parameters measured manually at admission.

## Conclusions

Central obesity was associated with poor IHCA outcomes, after adjusting for the effects of BMI. Even if patients were classified as underweight or normal weight, those with central obesity may still have worse outcomes. Therefore, haemodynamic-directed CPR may be preferable to a “one-size-fits-all” resuscitation algorithm and could be possibly applied for CPR in ICUs.

## Data Availability

The data that support the findings of this study are available on request from the corresponding author, Wen-Jone Chen.
